# Social isolation and the risk of Parkinson disease in the UK biobank study

**DOI:** 10.1038/s41531-024-00700-7

**Published:** 2024-04-08

**Authors:** Tingting Geng, Yaqi Li, Yinshun Peng, Xiao Chen, Xinming Xu, Jian Wang, Liang Sun, Xiang Gao

**Affiliations:** 1 Department of Nutrition and Food Hygiene, School of Public Health, Institute of Nutrition, Fudan University, Shanghai, China; 2 Department of Neurology and National Research Center for Aging and Medicine & National Center for Neurological Disorders, State Key Laboratory of Medical Neurobiology, Huashan Hospital, Fudan University, Shanghai, China

**Keywords:** Risk factors, Parkinson's disease

## Abstract

Parkinson disease (PD) has become one of the most rapidly growing causes of disability among the older population and social isolation is a major concern in the PD community. However, the relationship between social isolation and future risk of PD remains unclear. This study included 192,340 participants aged 60 or older who were free of dementia and PD at baseline from the UK Biobank study. Social isolation was measured using a composite score derived from three questions on number in household, frequency of friend/family visits, and leisure/social activities. Incident PD cases were identified through electronic health records. Multivariable-adjusted Cox regression models were used to compute the hazard ratio (HR) and 95% confidence interval (CI). Among the 192,340 participants (mean [standard deviation] age, 64.2 [2.9] years; 103,253 [53.7%] women), 89,075 (46.3%) participants were in the least isolated group and 26,161 (13.6%) were in the most isolated group. Over a median follow-up of 12.5 years, 2048 incident PD cases were documented. Compared to the least isolated group, the multivariable-adjusted HRs (95% CIs) for PD were 1.00 (0.91−1.10) for the moderately isolated group and 1.19 (1.05−1.36) for the most isolated group (*P*-_trend_ = 0.04). The observed association was independent of the genetic susceptibility to PD and consistent in subgroup analyses. Social isolation was associated with a higher risk of PD regardless of genetic risk. Our findings highlighted the importance of developing screening and intervention strategies for social isolation among older adults to reduce the risk of PD.

## Introduction

Parkinson disease (PD) is a chronic progressive neurodegenerative disease characterized by symptoms such as tremor, rigidity, and bradykinesia, which is the second most common neurodegenerative disease^1^. In addition, PD has become one of the most rapidly growing causes of disability among the older population in the world, with a significant economic burden borne by both individuals and health systems^2^. Although symptomatic therapies can help patients to maintain good quality of life for several years, therapies to modify or reverse the disease do not exist. Therefore, to identify the modifiable risk factors is paramount to prevent or delay the development of PD.

Social isolation, defined as a lack of meaningful social connections^3^, is an increasing public health concern among older adults. In the United States, it is estimated that over 20% of the older adults do not have social connections with friends or family^4^. Experience of social isolation has been linked to a higher risk of depression, dementia, cardiovascular disease, and premature death^5–10^. Among patients with PD, social isolation was associated with worsened severity, decreased quality of life and elevated mortality rates^11,12^. However, the relationship between social isolation and the risk of developing PD remains unclear. We hypothesized that social isolation could be a risk factor of PD in the present study.

To address the research gaps, we examined the prospective association between different levels of social isolation and risk of PD, leveraging data from the UK Biobank study, which represents one of the largest community-based cohort studies. Additionally, as PD is influenced by both genetic and environment risk factors^13,14^, we also investigated whether the association between social isolation and PD could be modified by genetic determinants of PD.

## Results

### Baseline characteristics

Among the 192,340 participants included in the present analysis (53.7% women), the mean age [SD] was 64.2 [2.9] years. Of all the study participants, 26,161 (13.6%) were defined as the most isolated, 77,104 (40.1%) were in the moderately isolated category and 89,075 (46.3%) were in the least isolated category. Distributions of baseline characteristics of the study population according to social isolation status are presented in Table 1. Compared with the least isolated group, participants in the most isolated group were more likely to be men, deprived, non-White, current smokers, never drinkers, and sleep deprived. They also tended to have a higher prevalence of pre-existing hypertension, diabetes, cardiovascular disease, and chronic kidney disease.

**Table 1:** Baseline characteristics according to social isolation in the UK Biobank study

	Social isolation			Effect size^a^	*P*-value
Characteristic	Least isolated	Moderately isolated	Most isolated		
Number of participants	89,075 (46.3)	77,104 (40.1)	26,161 (13.6)		
Age, year	64.3 (2.8)	64.2 (2.9)	64.1 (2.9)	42.7	0.002
Sex				211.4	< 0.001
Women	47,515 (53.3)	42,612 (55.3)	13,126 (50.2)		
Men	41,560 (46.7)	34,492 (44.7)	13,035 (49.8)		
Education				97.4	< 0.001
College or university degree	64,304 (72.2)	56,746 (73.6)	19,492 (74.5)		
Others	23,768 (26.7)	19,375 (25.1)	6323 (24.2)		
Missing	1003 (1.1)	983 (1.3)	346 (1.3)		
Townsend deprivation index	−2.08 (2.62)	−1.36 (3.03)	−0.43 (3.42)	3559.0	< 0.001
Ethnicity				259.7	< 0.001
White	86,915 (97.6)	74,671 (96.8)	25,038 (95.7)		
Others	2160 (2.4)	2433 (3.2)	1123 (4.3)		
Body mass index, kg/m^2^	27.5 (4.3)	27.6 (4.6)	27.8 (5.0)	73.5	< 0.001
Smoking status				2200.0	< 0.001
Never	46,129 (51.8)	37,816 (49.1)	11,637 (44.5)		
Past	37,515 (42.1)	32,023 (41.5)	10,598 (40.5)		
Current	5095 (5.7)	6863 (8.9)	3767 (14.4)		
Missing	336 (0.4)	402 (0.5)	159 (0.6)		
Alcohol status				3600.0	< 0.001
Never or special occasions	13,939 (15.7)	17,375 (22.5)	8336 (31.9)		
Monthly to weekly	52,790 (59.3)	42,152 (54.7)	12,344 (47.2)		
Daily drinkers	22,312 (25.1)	17,519 (22.7)	5454 (20.9)		
Missing	34 (0.04)	58 (0.08)	27 (0.1)		
Sleep duration, hours/day				1100.0	< 0.001
≤6	18,121 (20.3)	18,800 (24.4)	7497 (28.7)		
7-8	61,606 (69.2)	50,368 (65.3)	15,708 (60.0)		
≥9	8970 (10.1)	7399 (9.6)	2703 (10.3)		
Missing	382 (0.4)	537 (0.7)	253 (1.0)		
History of cardiovascular disease, yes	9261 (10.4)	8492 (11.0)	3337 (12.8)	115.6	< 0.001
History of diabetes, yes	6223 (7.0)	6599 (8.6)	2893 (11.1)	472.8	< 0.001
History of hypertension, yes	63,390 (71.2)	55,321 (71.8)	19,065 (72.9)	30.0	< 0.001
History of chronic kidney disease, yes	3225 (3.6)	3064 (4.0)	1222 (4.7)	61.1	< 0.001
History of cancer, yes	9590 (10.8)	8504 (11.0)	2897 (11.1)	3.74	0.15

Data are presented as mean (SD) or *n* (%).

^a^Effect sizes were *F*-values for continuous variables and χ² for categorical variables.

### Association of social isolation and risk of PD

During a median of 12.5 (IQR,11.7–13.2) years of follow-up, 2048 incident PD events were documented. Compared with the least isolated group, the HRs (95% CIs) of PD were 1.01 (0.92–1.11) for moderately isolated group and 1.23 (1.08–1.40) for most isolated group (*P*-_trend_ = 0.01) after adjusting for the social demographic characteristics in Model 1. The corresponding estimates were slightly attenuated to 1.00 (0.91–1.10) and 1.19 (1.05–1.36) after the adjustment for lifestyle factors and comorbidities in Model 2 (*P*-_trend_ = 0.04; Table 2).

**Table 2:** HR (95% CI) of Parkinson disease according to social isolation

	Social isolation			
	Least isolated	Moderately isolated	Most isolated	*P*-trend
Cases/person-years	938/1,083,152	788/925,829	322/308,040	
HR (95% CI)^a^	1.00	1.01 (0.92–1.11)	1.23 (1.08–1.40)	0.01
HR (95% CI)^b^	1.00	1.00 (0.91–1.10)	1.19 (1.05–1.36)	0.04

^a^Adjusted for age at recruitment (continuous, years), sex (men, women), education (college or university degree, others), Townsend Deprivation Index (continuous), and ethnicity (White, others).

^b^Adjusted for age at recruitment (continuous, years), sex (men, women), education (college or university degree, others), Townsend Deprivation Index (continuous), ethnicity (White, others), body mass index (continuous, kg/m^2^), alcohol intake (never or special occasions, monthly to weekly, daily), smoking status (never, past, current), sleep duration (≤6, 7–8, ≥9 hours/day), and history of hypertension, cardiovascular disease, diabetes, cancer, and chronic kidney disease (yes, no).

The weighted GRS was significantly associated with PD. With each unit increment of the weighted GRS, the HR (95% CI) of PD was 1.07 (1.05–1.08) with the adjustment for age and sex (Supplementary Table 3). In addition, for each additional unit increase in the social isolation score, the fully adjusted HR (95% CI) of PD was 1.08 (1.02–1.15) (Supplementary Table 4).

### Secondary analyses and sensitivity analyses

In the stratified analysis by genetic susceptibility to PD, comparing the most isolated with the least isolated groups, the HR (95% CI) of PD was 1.28 (1.07–1.52) among individuals at high genetic risk of PD. In contrast, the association was significantly attenuated and not significant among the participants at low genetic risk of PD, although *P* for interaction did not reach statistical significance (*P*-_interaction_ = 0.51; Table 3).

**Table 3:** HR (95% CI) of Parkinson’s disease according to social isolation stratified by genetic risk score for Parkinson disease

	Social isolation			
Genetic risk score for Parkinson disease	Least isolated	Moderately isolated	Most isolated	*P*-trend
Low ( < median)				
Cases/person-years	391/547,230	325/466,031	123/154,158	
HR (95% CI)	1.00	0.98 (0.84–1.13)	1.07 (0.86–1.31)	0.73
High ( ≥ median)				
Cases/person-years	524/507,908	438/429,004	180/139,955	
HR (95% CI)	1.00	1.01 (0.89–1.15)	1.28 (1.07–1.52)	0.02
* P*-interaction	0.51			

HRs were adjusted for age at recruitment (continuous, years), sex (men, women), education (college or university degree, others), Townsend Deprivation Index (continuous), body mass index (continuous, kg/m^2^), alcohol intake (never or special occasions, monthly to weekly, daily), smoking status (never, past, current), sleep duration (≤6, 7–8, ≥9 hours/day), and history of hypertension, cardiovascular disease, diabetes, cancer, chronic kidney disease (yes, no), genetic batches and the first 10 principal component analysis.

**Fig. 1:**
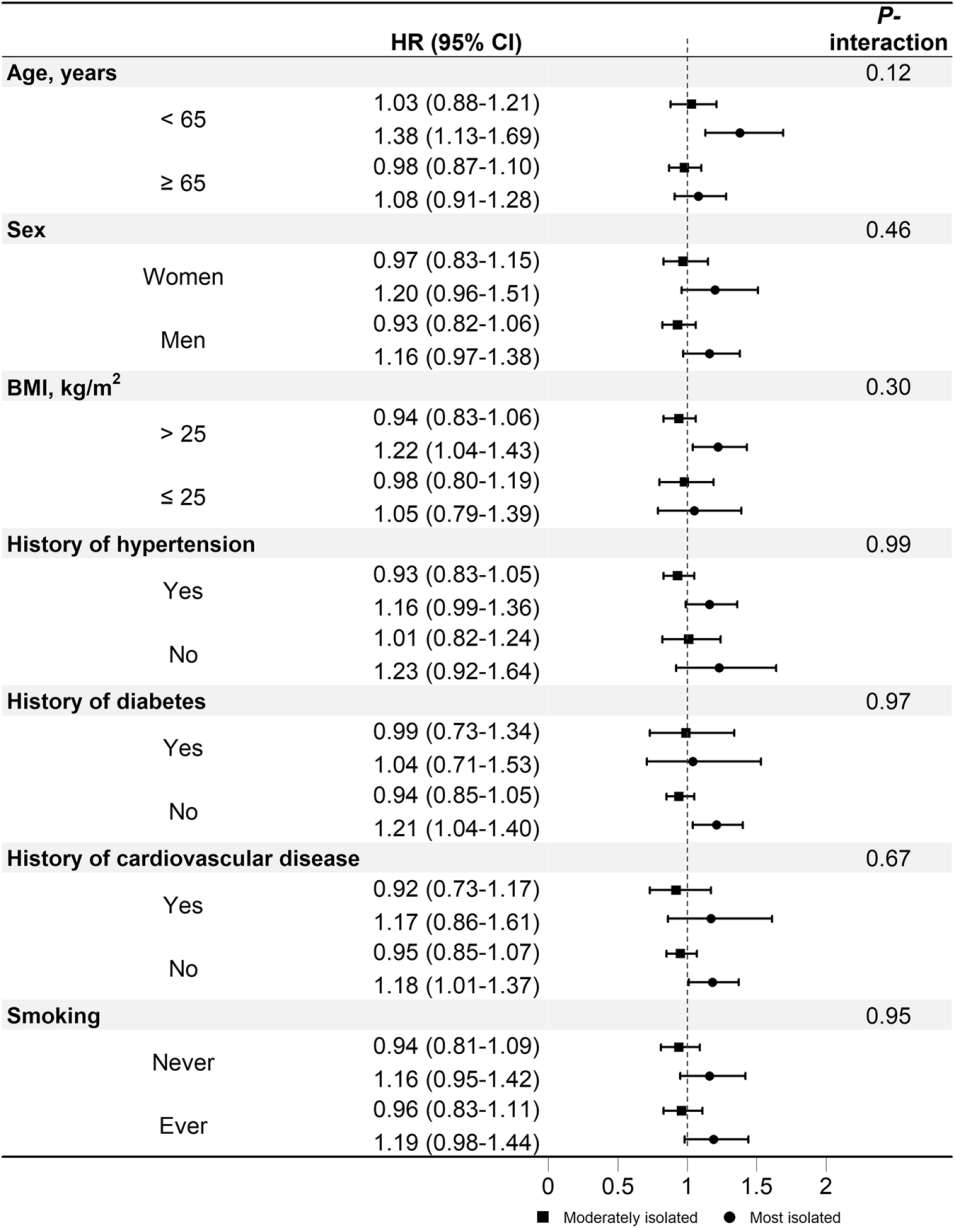
Subgroup analysis of social isolation in relation to the risk of Parkinson disease among individuals of 60 years or older. HRs were adjusted for age at recruitment (continuous, years), sex (men, women), education (college or university degree, others), Townsend Deprivation Index (continuous), ethnicity (White, others), body mass index (continuous, kg/m^2^), alcohol intake (never or special occasions, monthly to weekly, daily), smoking status (never, past, current), sleep duration (≤6, 7–8, ≥9 hours/day), and history of hypertension, cardiovascular disease, diabetes, cancer, and chronic kidney disease (yes, no), except for the stratified factors. The hazard ratios are indicated by the circles and squares, and the 95% confidence intervals are shown by the error bars.

In the component analysis, frequency of friend/family visits was significantly associated with a higher risk of PD. Compared to individuals having friend/family visits about once a week to almost daily, the HR (95% CI) of PD was 1.12 (1.00–1.25) for those with no friends/family to about once a month (Supplementary Table 5). In addition, we did not find significant heterogeneity in the risk estimates of social isolation, and the stratified factors including age, sex, smoking status, BMI, history of hypertension, diabetes, and cardiovascular disease on the risk of PD (*Ps*-_interaction_ > 0.05; Fig. 1).

Our results remained robust when we excluded participants with less than two years of follow-up (Supplementary Table 6), or on the dataset with multiple imputation method (Supplementary Table 7), or with the additional adjustment for C-reactive protein, loneliness, and physical activity (Supplementary Table 8).

## Discussion

Our data from this population-based study of 192,340 participants aged 60 years or older showed that the most social isolation was associated with a higher risk of developing PD. The observed association was independent of the social demographic factors, lifestyles, chronic conditions, and the genetic susceptibility to PD.

Our results complement the body of work on associations of social isolation with a broad spectrum of health outcomes. In the UK Biobank study and Million Women Study with 938,558 participants, social isolation was associated with a higher risk of coronary heart disease (HR: 1.86; 95% CI: 1.63–2.12) and stroke events (HR: 1.91; 95% CI: 1.48–2.46) that resulted in death without an associated hospital admission^7^. Social isolation was also associated with a higher risk of incident heart failure in the UK Biobank study (most vs. least social isolation, HR: 1.17; 95% CI: 1.11–1.23), and the observed association was independent of the genetic risk of heart failure^6^. Further, the National Health and Aging Trends Study representing 5,705,675 survey-weighted intensive care unit hospitalizations, assessed the social isolation using 6 questions corresponding to domains including social connectedness, contacts with family and friends, membership in a religious organization, and participation in other community groups. The study found that a higher score of social isolation was associated with a higher risk of post-intensive care unit disability and mortality^9^. In addition, social isolation was associated with 1.26-fold increased risk of developing all-cause dementia and individuals who were socially isolated had lower volumes of gray matter^15^.

The component analysis showed that the frequency of friend/family visits was significantly associated with the risk of developing PD, which suggests that among the metrics of social isolation, the frequency of friend/family visits may be an independent risk factor of PD. Similarly, a cross-sectional study also showed that in individuals with PD, those with more friends had 21% fewer symptoms of PD than those with no friends^11^. However, future studies with prospective design and more cases are needed to clarify the associations between items of social isolation and risk of development and progression of PD.

Although the exact mechanism underlying the relationship between social isolation and risk of PD remains to be further elucidated, we speculated several potential mechanisms to explain the observed association. First, social isolation may contribute to PD development through dysregulations of the hypothalamic-pituitary-adrenal axis and the sympathetic nervous system^16,17^. Further, social isolation was associated with increased systemic and neuro-inflammation and oxidative stress, which play a critical role in the development and progression of PD^18–20^. In addition, social disconnection can induce mitochondrial dysfunction, and increasing evidence has highlighted the mitochondrial dysfunction as a critical driver of PD pathology^21–23^. Finally, social isolation can modulate social inequality and further restrict people from seeking social support and health care resources, which may lead to poor management of risk factors and prodromal symptoms of PD^24^. Nevertheless, more studies are warranted to further illuminate the potential mechanisms through which social isolation plays a role in the development of PD.

PD is a rising public health issue. The number of people who are living with PD and the resulting disability and deaths are increasing faster than any other neurodegenerative disorders^25^. It is well-known that clinical PD is preceded by a long prodromal period before the onset of the classic motor features^26^. Therefore, it is paramount to identify the risk factors and eliminate/management the risk factors for PD prevention. Social isolation is a major issue among older adults. The National Health and Aging Trends Study showed that 24% of the adults aged 65 or older reported to be socially isolated in 2011^4^. A latest meta-analysis including 30 investigations during the COVID-19 reported that the prevalence of social isolation was 31.2% among older adults^27^. Our findings, if confirmed, may contribute towards the scientific basis for the development of public health strategies that targeted on eliminating and improving social isolation status for PD prevention among the older adults.

The strengths of this study include its prospective study design, large sample size, long-term follow-up, and meticulous adjustment for a wide range of potential confounding factors. However, our results should be interpreted in the context of several potential limitations. First, the self-reported data on social isolation was subject to measurement error and some misclassification was inevitable. However, given the prospective study design, such misclassification could have underestimated the true associations between social isolation and risk of PD. In addition, the questions used to derive social isolation in the UK Biobank have not been validated; however, social isolation in the UK Biobank has been associated with multiple health outcomes in previous studies^6,7,10,28^. Second, some early staged cases of PD may not be captured because the incident PD cases were ascertained only through electronic health records. This approach may lead to misclassification of cases. Third, the self-reported and one-time assessment of covariates may result in misclassification bias. Fourth, majority of the study population was White, thus limiting the generalizability of our findings to other ethnic groups. Finally, due to the nature of observational study design, residual confounding cannot be completely ruled out.

## Methods

### Study population

The UK Biobank is a large population-based prospective cohort study including more than 500,000 participants (aged 37–73 years) from 22 assessment centers across the UK from Mar 2006 to Dec 2010. Participants provided information on socio-demographics, habitual diet, lifestyle factors, and medical history through touch-screen questionnaires and face-to-face interviews at recruitment. After interview, participants underwent a standardized portfolio of physical measurements including anthropometric data, blood pressure indices, etc. Blood, urine, and saliva samples were also collected among all the participants at baseline. Details of the study were presented previously^29,30^. The UK Biobank study was approved by the National Information Governance Board for Health and Social Care in England and Wales, and the Community Health Index Advisory Group in Scotland and the North West Multicenter Research Ethics Committee. All participants gave written informed consent.

A total of 192,340 participants were included in the current analysis after excluding individuals with a previous diagnosis of PD and dementia (*n* = 839), those aged < 60 years old (*n* = 284,945), or those with missing values on social isolation items (*n* = 24,284). The flowchart for the selection of the study population is presented in Supplementary Figure 1.

### Assessment of social isolation

Social isolation was assessed by asking three questions: (1) “Including yourself, how many people live in your household? Include those who usually live in the house such as students living away from home during term time, and partners in the armed forces or in professions such as pilots” Participants received 1 point if they answered living alone; (2) “How often do you visit friends or family or have them visit you?” Participants were assigned 1 point if they answered less than one friend or family visit per month; and (3) “Which of the following (leisure or social activities) do you engage in once a week or more often? You may select more than one” Participants reported not participating in any social activities received 1 point. The final score of social isolation ranged 0–3 and was further classified into least isolation (0 point); moderate isolation (1 point); and most isolation (≥2 points).

### Weighted genetic risk score construction

Details of the design of genotyping, quality control and imputation procedures were described previously^31^. In the gene and environmental interaction analysis section, we further excluded the participants who did not have European ancestry, exhibited discrepancies between genetic sex and self-reported sex, and those had excessive heterozygosity or high levels of missingness.

We selected 44 single nucleotide polymorphisms (SNPs) associated with PD at genome-wide significance (*p* < 5 × 10^−8^) among individuals of European ancestry^32^, which is present in Supplementary Table 1. All the 44 SNPs were available in the UK Biobank. Individual SNPs were coded as 0, 1, or 2 based on the number of risk alleles. A weighted genetic risk score (GRS) for each participant was calculated by the widely used formula: weighted GRS = (β_1_ × SNP_1_ + β_2_ × SNP_2_ + β_3_ × SNP_3_ + … +β_n_ × SNP_n_) / (average of the β coefficients). The weighted GRS followed a normal distribution, ranging from 13.8 to 47.9 as shown in Supplementary Figure 2, and a higher score indicated a higher genetic predisposition to PD. The GRS score was categorized into low (<median) and high (≥median) groups for further analysis.

### Ascertainment of incident PD

Incident PD cases were identified using the algorithm method developed by the UK Biobank, with a positive predictive values of 91%^33^. These cases are determined by combining coded data from multiple sources, including participants’ self-reported medical conditions, operations, and medications collected during data collection at baseline. Additionally, data from hospital admissions and death registries are also linked to provide a comprehensive record in PD cases. Details of the definition are present in Supplementary Table 2. Each participant’s person-year was calculated from the date of recruitment to the date reported for PD diagnosis, death, loss to follow-up, or end of the follow-up (10^th^ Oct 2021), whichever occurred first.

### Assessment of the covariates

Data on age, sex, ethnicity, education, sleep duration, smoking status, and alcohol intake were acquired at baseline through a touchscreen questionnaire. Townsend deprivation index (TDI) is a composite measure of area-level socio-economic deprivation, with higher scores indicating greater deprivation^34^. Body mass index (BMI) was calculated by dividing body weight in kilograms by the square of height in meters. Total physical activity including walking and moderate and vigorous intensity activities, in metabolic equivalent minutes per week (MET-min/week) was computed from the International Physical Activity Questionnaire (IPAQ). Physical activity levels <10 min/day was recoded to 0^35^. Pre-existing hypertension was defined based on self-reported physician diagnosis, use of anti-hypertensive medications, essential hypertension cases based on ICD-10 codes (I10), or blood pressure measurements of 140/90 mm Hg or higher. Diabetes was defined by self-reported diagnosis, use of insulin or hypoglycemic drugs, diabetes-related ICD-10 codes (E10-14), fasting glucose levels of ≥7.0 mmol/L, random glucose levels of ≥11.1 mmol/L, or HbA1c levels of ≥48 mmol/mol (6.5%). Prevalent cardiovascular disease cases including ischemic heart disease, stroke, and heart failure were identified using ICD-10 codes (I20-I25, I50, I60-I64). Prevalent cancer cases were self-reported, and prevalent chronic kidney disease cases were defined as estimated glomerular filtration rate (eGFR) <60 mL/min per 1.73 m^2^. Loneliness was assessed using the 2 questions from the revised loneliness index: 1) “Do you often feel lonely?” and 2) “How often are you able to confide in someone close to you?”^36^

### Statistical analysis

Missing values for continuous variables (0.09% for TDI, and 0.5% for BMI) were imputed using median values, and missing values for categorical variables (0.06% for alcohol intake, 0.5% for smoking, 1.2% for education, and 0.6% for sleep duration) were imputed using the indicator approach. The differences in baseline characteristics by social isolation were examined using the chi-squared test for categorical variables and ANOVA test for continuous variables.

We used multivariable Cox proportional hazards regression models to compute hazard ratios (HRs) and 95% confidence intervals (CIs) for the association between social isolation and risk of PD. Schoenfeld residuals method showed the assumption of proportional hazards was not violated. In Model 1, we adjusted for age (continuous, years), sex (men, women), ethnicity (White, non-White), TDI (continuous), and education (college and above, others). In Model 2, we further adjusted for smoking status (never, former, current), alcohol intake (never or special occasions/occasionally, monthly to weekly, daily), BMI (continuous, kg/m^2^), sleep duration (≤6, 7–8, ≥9 hours/day), and history of hypertension, cardiovascular disease, diabetes, cancer, and chronic kidney disease (yes, no). We chose variables for adjustment beforehand, considering their availability and general understanding of PD’s risk factors^37,38^.

Further, we stratified the analysis by the GRS of PD (low vs. high) and tested the interaction between social isolation and the GRS using the likelihood ratio test by including an interaction term in the multivariable-adjusted model. In the models with GRS, genotyping batch, and the first 10 principal component of ancestry were additionally adjusted based on Model 2. We also tested the association between the social isolation score and risk of PD. Further, we stratified the analyses by age (<65, ≥65 years), sex (men, women), BMI (<25, ≥25 kg/m^2^), smoking status (never, ever smoking), history of hypertension (yes, no), history of diabetes (yes, no), and history of cardiovascular disease (yes, no). The interactions of social isolation and the stratified factors on the risk of PD were also tested. In addition, we investigated the associations of individual social isolation items with risk of PD with mutual adjustment for the other social isolation items.

We performed several sensitivity analyses to test the robustness of our findings. First, to minimize the potential confounding effect of preclinical PD on the observed associations, we repeated the analyses after excluding cases that occurred within the first two years of follow-up. Second, we performed the analysis using the multiple imputation by chained equations with 5 imputations. Finally, to determine whether the observed association between social isolation and risk of PD could be explained by systemic inflammation, loneliness, or physical activity, we further adjusted for circulating C-reactive protein (continuous, mg/L), loneliness, and total MET-mins per week based on Model 2.

All analyses were performed in Stata statistical software, release 15.1 (StataCorp LP, College Station, Texas), and *P*-value < 0.05 was considered as statistically significant.

### Reporting summary

Further information on research design is available in the Nature Research Reporting Summary linked to this article.

### Supplementary information

Supplementary materials

Reporting summary

## Data Availability

The UK Biobank data can be retrieved by applying to the UK Biobank, www.ukbiobank.ac.uk/. This research has been conducted using the UK Biobank Resource under Application Number 96083.

## Code availability

Codes are available from the corresponding author upon reasonable request.
