# Supplementary material for: Social isolation and the risk of Parkinson disease in the UK biobank study
Source: NPJ Parkinsons Dis. 2024 Apr 8;10:79. doi: 10.1038/s41531-024-00700-7 (PMC11001945; doi:10.1038/s41531-024-00700-7)
Supplement: Supplementary file 1 — Supplementary materials [file 41531_2024_700_MOESM1_ESM.docx]

**Supplementary materials**

**Supplementary Table 1.** SNPs used to generate the genetic risk score for Parkinson disease

| **SNP** | **Effect allele** | **EAF** | **p-value** | **OR** |
| --- | --- | --- | --- | --- |
| rs10797576 | T/C | 0.137 | 1.76 × 10^−10^ | 1.13 |
| rs10906923 | C/A | 0.306 | 2.37 × 10−8 | 0.93 |
| rs11060180 | G/A | 0.45 | 3.08 × 10−11 | 0.91 |
| rs11158026 | T/C | 0.307 | 2.88 × 10−10 | 0.91 |
| rs11343 | T/G | 0.454 | 1.46 × 10−9 | 1.07 |
| rs115185635 | C/G | 0.036 | 2.2 × 10−8 | 1.79 |
| rs11724635 | C/A | 0.437 | 4.26 × 10−17 | 0.89 |
| rs117896735 | A/G | 0.012 | 1.21 × 10−11 | 1.77 |
| rs12456492 | G/A | 0.332 | 2.15 × 10−11 | 1.10 |
| rs12497850 | G/T | 0.347 | 6.80 × 10−8 | 0.93 |
| rs12637471 | A/G | 0.219 | 5.38 × 10−22 | 0.84 |
| rs13294100 | T/G | 0.371 | 1.99 × 10−12 | 0.91 |
| rs14235 | A/G | 0.397 | 3.63 × 10−12 | 1.10 |
| rs143918452 | G/A | 0.996 | 2.25 × 10−7 | 0.68 |
| rs1474055 | C/T | 0.881 | 7.11 × 10−16 | 0.82 |
| rs1555399 | T/A | 0.544 | 5.70 × 10−16 | 1.15 |
| rs17649553 | T/C | 0.232 | 6.11 × 10−49 | 0.77 |
| rs199347 | G/A | 0.368 | 5.62 × 10−14 | 0.90 |
| rs2280104 | T/C | 0.367 | 9.06 × 10−7 | 1.06 |
| rs2414739 | G/A | 0.292 | 3.59 × 10−12 | 0.90 |
| rs2694528 | C/A | 0.115 | 1.69 × 10−11 | 1.15 |
| [rs2740594](https://www.ncbi.nlm.nih.gov/pmc/articles/PMC5812477/table/T2/?report=objectonly#TFN12) | A/G | 0.753 | 9.54 × 10−11 | 1.10 |
| rs329648 | T/C | 0.327 | 8.05 × 10−12 | 1.11 |
| rs34043159 | C/T | 0.352 | 3.83 × 10−8 | 1.07 |
| rs34311866 | C/T | 0.199 | 6.00 × 10−41 | 1.26 |
| rs353116 | T/C | 0.385 | 9.73 × 10−7 | 0.94 |
| rs356182 | G/A | 0.375 | 1.85 × 10−82 | 1.34 |
| rs35749011 | G/A | 0.976 | 6.10 × 10−23 | 0.57 |
| rs3793947 | A/G | 0.463 | 2.59 × 10−8 | 0.91 |
| rs4073221 | G/T | 0.132 | 3.02 × 10−9 | 1.11 |
| rs4653767 | C/T | 0.315 | 2.40 × 10−10 | 0.92 |
| rs4784227 | T/C | 0.265 | 8.29 × 10−8 | 1.08 |
| rs591323 | A/G | 0.293 | 3.17 × 10−8 | 0.91 |
| rs601999 | C/T | 0.699 | 8.03 × 10−9 | 0.93 |
| rs62120679 | t/C | 0.324 | 2.52 × 10−9 | 1.14 |
| rs6430538 | T/C | 0.488 | 3.35 × 10−19 | 0.88 |
| [rs6812193](https://www.ncbi.nlm.nih.gov/pmc/articles/PMC5812477/table/T1/?report=objectonly#TFN8) | T/C | 0.398 | 1.85 × 10−11 | 0.91 |
| rs76904798 | T/C | 0.132 | 4.86 × 10−14 | 1.16 |
| rs78738012 | C/T | 0.106 | 2.11 × 10−9 | 1.14 |
| rs8005172 | T/C | 0.424 | 1.20 × 10−9 | 1.08 |
| rs8118008 | A/G | 0.596 | 2.32 × 10−8 | 1.11 |
| rs823118 | C/T | 0.467 | 1.96 × 10−16 | 0.89 |
| rs9275326 | T/C | 0.114 | 5.81 × 10−13 | 0.80 |
| rs9468199 | A/G | 0.172 | 3.44 × 10−13 | 1.12 |

EAF, effect allele frequency; SNP, single nucleotide polymorphism; OR, odds ratio.

**Supplementary Table 2.** Ascertainment for Parkinson disease cases in the UK Biobank study

| **ICD-9** | **ICD-10** | **Self-reported in UK Biobank (field ID)** |
| --- | --- | --- |
| 3320, 3321, 3330 | G20, G21, G21.0, G21.1, G21.2, G21.3, G21.4, G21.8, G21.9, G22, G23.0, G23.1, G23.2, G23.3, G23.8, G23.9, G25.9, G26, G90.3 | Field 20002  Code 1262 |

**Supplementary Table 3.** Associations of the genetic risk score with the risk of Parkinson disease

|  | **Parkinson disease** | |
| --- | --- | --- |
|  | **HR (95%CI)^1^** | ***P*-value** |
| Genetic risk score, per unit | 1.07 (1.05-1.08) | < 0.001 |

^1^ Adjusted for age at recruitment (continuous, years), and sex (men, women).

**Supplementary Table 4.** HR (95% CI) of Parkinson disease according to the social isolation score

|  | **Parkinson disease** | |
| --- | --- | --- |
|  | **HR (95%CI)^1^** | ***P*-value** |
| Social isolation score (0-3), per unit | 1.08 (1.02-1.15) | < 0.001 |

^1^ Adjusted for age at recruitment (continuous, years), sex (men, women), education (college or university degree, others), Townsend Deprivation Index (continuous), ethnicity (White, others), body mass index (continuous, kg/m^2^), alcohol intake (never or special occasions, monthly to weekly, daily), smoking status (never, past, current), sleep duration (≤6, 7–8, ≥9 hours/day), and history of hypertension, cardiovascular disease, diabetes, cancer, and chronic kidney disease (yes, no).

**Supplementary Table 5.** HR (95% CI) of Parkinson disease according to individual social isolation items

| **Items of social isolation** | **HR (95% CI)^1^** | **P-value** |
| --- | --- | --- |
| **Number in household** |  |  |
| More than one | 1.00 |  |
| One | 1.05 (0.95-1.17) | 0.34 |
| **Frequency of friend/family visits** |  |  |
| About once a week to almost daily | 1.00 |  |
| No friends/family to about once a month | 1.12 (1.00-1.25) | 0.047 |
| **Leisure/social activities** |  |  |
| Yes | 1.00 |  |
| No | 1.08 (0.98-1.19) | 0.14 |

^1^ Adjusted for age at recruitment (continuous, years), sex (men, women), education (college or university degree, others), Townsend Deprivation Index (continuous), ethnic (White, others), body mass index (continuous, kg/m^2^), alcohol intake (never or special occasions, monthly to weekly, daily), smoking status (never, past, current), sleep duration (≤6, 7–8, ≥9 hours/day), and history of hypertension, cardiovascular disease, diabetes, cancer, chronic kidney disease (yes, no), and also mutually adjusted for the other items of social isolation.

**Supplementary Table 6.** HR (95% CI) of Parkinson’s disease according to social isolation after excluding the cases occurring within the first two years of follow up

|  |  | **Social isolation** | |  |
| --- | --- | --- | --- | --- |
|  | **Least isolated** | **Moderately isolated** | **Most isolated** | **P-trend** |
| Cases/person-years | 914/1,083,120 | 761/925,802 | 309/308,023 |  |
| HR (95% CI) ^1^ | 1.00 | 0.99 (0.90-1.09) | 1.18 (1.04-1.35) | 0.06 |

^1^ Adjusted for age at recruitment (continuous, years), sex (men, women), education (college or university degree, others), Townsend Deprivation Index (continuous), ethnicity (White, others), body mass index (continuous, kg/m^2^), alcohol intake (never or special occasions, monthly to weekly, daily), smoking status (never, past, current), sleep duration (≤6, 7–8, ≥9 hours/day), and history of hypertension, cardiovascular disease, diabetes, cancer, and chronic kidney disease (yes, no).

**Supplementary Table 7.** HR (95% CI) of Parkinson disease according to social isolation using multiple imputation method

|  |  | **Social isolation** | |  |
| --- | --- | --- | --- | --- |
|  | **Least isolated** | **Moderately isolated** | **Most isolated** | **P-trend** |
| HR (95% CI) ^1^ | 1.00 | 1.00 (0.91-1.10) | 1.20 (1.05-1.37) | 0.03 |

^1^ Adjusted for age at recruitment (continuous, years), sex (men, women), education (college or university degree, others), Townsend Deprivation Index (continuous), ethnic (White, others), body mass index (continuous, kg/m^2^), alcohol intake (never or special occasions, monthly to weekly, daily), smoking status (never, past, current), sleep duration (≤6, 7–8, ≥9 hours/day), and history of hypertension, cardiovascular disease, diabetes, cancer, and chronic kidney disease (yes, no).

**Supplementary Table 8.** HR (95% CI) of Parkinson disease according to social isolation

|  |  | **Social isolation** | |  |
| --- | --- | --- | --- | --- |
|  | **Least isolated** | **Moderately isolated** | **Most isolated** | **P-trend** |
| HR (95% CI) ^1^ | 1.00 | 1.00 (0.91-1.10) | 1.19 (1.05-1.36) | 0.04 |
| HR (95% CI) ^2^ | 1.00 | 1.00 (0.91-1.10) | 1.19 (1.05-1.36) | 0.04 |
| HR (95% CI) ^3^ | 1.00 | 0.99 (0.90-1.09) | 1.18 (1.04-1.35) | 0.05 |

^1^ Adjusted for age at recruitment (continuous, years), sex (men, women), education (college or university degree, others), Townsend Deprivation Index (continuous), ethnic (White, others), body mass index (continuous, kg/m^2^), alcohol intake (never or special occasions, monthly to weekly, daily), smoking status (never, past, current), sleep duration (≤6, 7–8, ≥9 hours/day), and history of hypertension, cardiovascular disease, diabetes, cancer, chronic kidney disease (yes, no), and C-reactive protein (continuous, mg/L).

**^2^** Adjusted for age at recruitment (continuous, years), sex (men, women), education (college or university degree, others), Townsend Deprivation Index (continuous), ethnic (White, others), body mass index (continuous, kg/m^2^), alcohol intake (never or special occasions, monthly to weekly, daily), smoking status (never, past, current), sleep duration (≤6, 7–8, ≥9 hours/day), and history of hypertension, cardiovascular disease, diabetes, cancer, chronic kidney disease and loneliness (yes, no).

^3^ Adjusted for age at recruitment (continuous, years), sex (men, women), education (college or university degree, others), Townsend Deprivation Index (continuous), ethnic (White, others), body mass index (continuous, kg/m^2^), alcohol intake (never or special occasions, monthly to weekly, daily), smoking status (never, past, current), sleep duration (≤6, 7–8, ≥9 hours/day), and history of hypertension, cardiovascular disease, diabetes, cancer, chronic kidney disease and physical activity (MET-min/week).


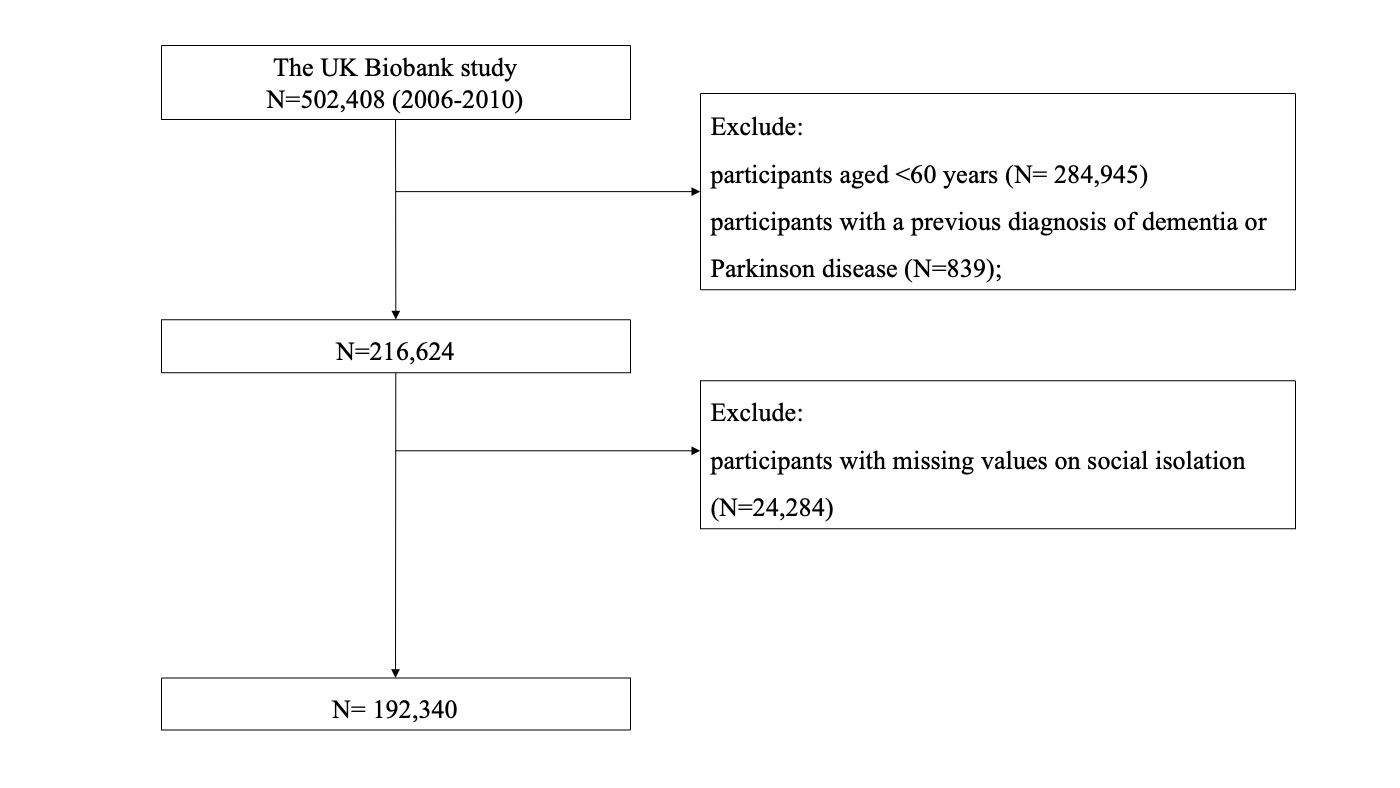


**Supplementary Figure 1.** Flowchart for the selection of the study population


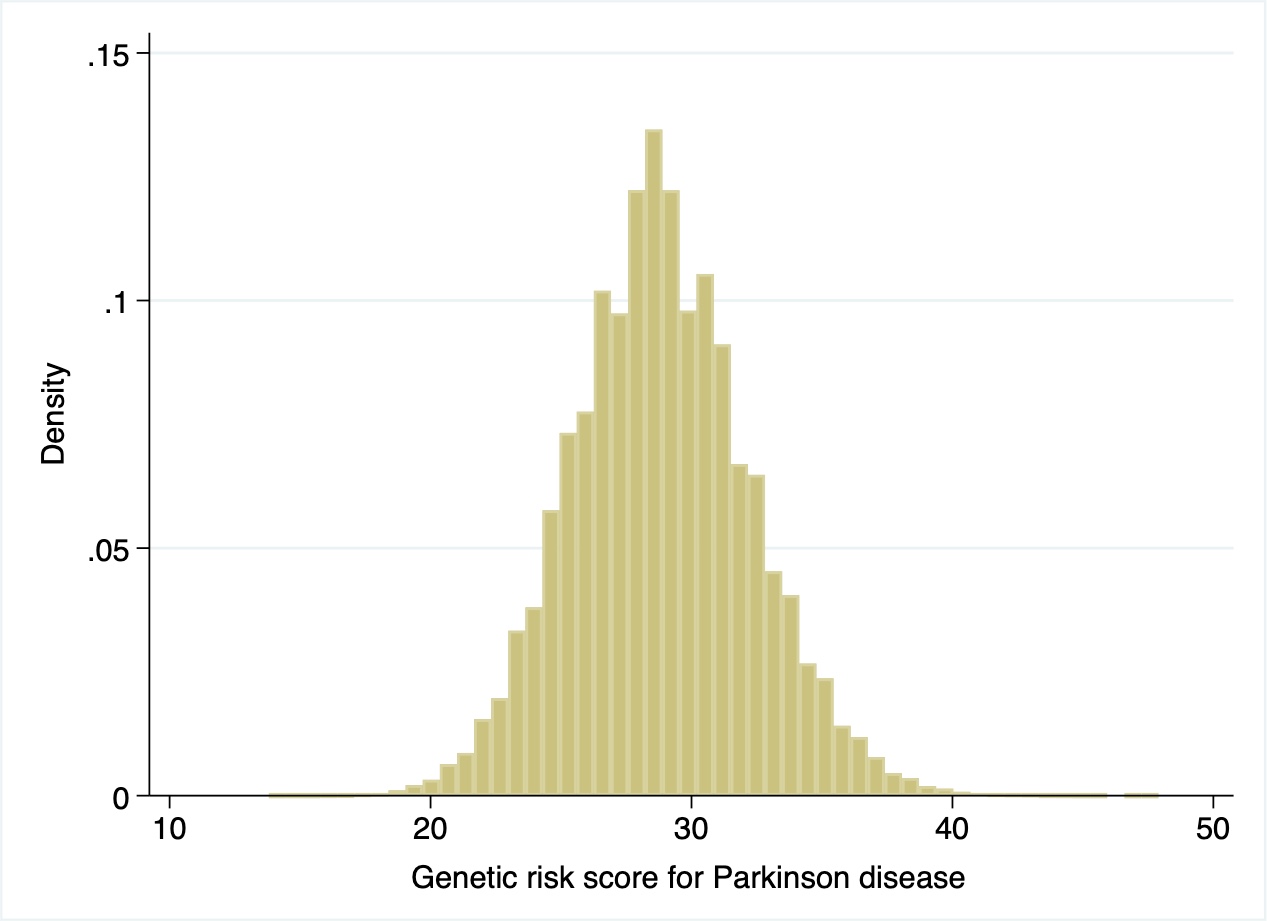


**Supplementary Figure 2.** Distribution of the genetic risk score for Parkinson disease
